# Hormone comparison between right and left baleen whale earplugs

**DOI:** 10.1093/conphys/coaa055

**Published:** 2020-06-24

**Authors:** Danielle D Crain, Amanda Thomas, Farzaneh Mansouri, Charles W Potter, Sascha Usenko, Stephen J Trumble

**Affiliations:** 1 Department of Biology, Baylor University, 101 Bagby Ave. Waco TX 76706, USA; 2 Department of Environmental Science, Baylor University, 101 Bagby Ave. Waco TX 76706, USA; 3 Department of Vertebrate Zoology, Smithsonian Institution National Museum of Natural History, PO Box 37012 SI Bldg, Room 153, MRC 010, Washington, DC 20013 USA; 4 Department of Chemistry and Biochemistry, Baylor University, 101 Bagby Ave. Waco TX 76706, USA

**Keywords:** Baleen whale, earplug, earwax, hormone baseline, validation, *Z*-score

## Abstract

Marine animals experience additional stressors as humans continue to industrialize the oceans and as the climate continues to rapidly change. To examine how the environment or humans impact animal stress, many researchers analyse hormones from biological matrices. Scientists have begun to examine hormones in continuously growing biological matrices, such as baleen whale earwax plugs, baleen and pinniped vibrissae. Few of these studies have determined if the hormones in these tissues across the body of the organism are interchangeable. Here, hormone values in the right and left earplugs from the same individual were compared for two reasons: (i) to determine whether right and left earplug hormone values can be used interchangeably and (ii) to assess methods of standardizing hormones in right and left earplugs to control for individuals’ naturally varying hormone expressions. We analysed how absolute, baseline-corrected and Z-score normalized hormones performed in reaching these goals. Absolute hormones in the right and left earplugs displayed a positive relationship, while using *Z*-score normalization was necessary to standardize the variance in hormone expression. After *Z*-score normalization, it was possible to show that the 95% confidence intervals of the differences in corresponding lamina of the right and left earplugs include zero for both cortisol and progesterone. This indicates that the hormones in corresponding lamina of right and left earplugs are no different from zero. The results of this study reveal that both right and left earplugs from the same baleen whale can be used in hormone analyses after Z-score normalization. This study also shows the importance of *Z*-score normalization to interpretation of results and methodologies associated with analysing long-term trends using whale earplugs.

## Introduction

Understanding longitudinal variability of analyte (e.g. hormone) concentrations within various biological matrices is necessary for assessing physiological state or ecological interpretation in species, individuals or populations (Rolland *et al.*, 2005; Landys *et al.*, 2006; Jenkins *et al.*, 2014). Traditionally, researchers have relied on samples representative of a single time point from biological matrices (i.e. blubber biopsies, faecal sampling) to assess relative analyte changes in response to natural or anthropogenic activity (Rolland *et al.*, 2005; Houser *et al.*, 2011; Kellar *et al.*, 2015). However, single time-point samples reflect only recent exposures, limiting researchers’ capacity to estimate long-term temporal effects of environmental change. As field sampling methods advance and novel biological matrices are explored, assessing longitudinal samples from a single individual allows investigation of variability over longer time frames: an important method for interpreting potential cause and effect scenarios (Trumble *et al.*, 2013; Hunt *et al.*, 2017a; Lysiak *et al.*, 2018).

Recently, researchers have established that several biological matrices have the capacity to archive sustained longitudinal hormone data at the level of the individual spanning months to lifetimes, including baleen whale earwax, baleen plates and pinniped vibrissae (Trumble *et al.*, 2013, 2018; Hunt *et al.*, 2014; Karpovich *et al.*, 2018). In identifying potential sources of variability, the association between hormone concentrations within or between matrices should be examined to increase our understanding of how to interpret these data among individuals and through time.

Previous studies used baseline correction or *Z*-score normalization to correct for natural variation in hormones expression as a means to compare across multiple individuals (Fanson *et al.*, 2017; Trumble *et al.*, 2018). The steroid hormones targeted in this study are glucocorticoids (i.e. cortisol) and gonadocorticoids (i.e. progesterone) because of their ubiquitous use in studies of stress and reproduction. Cortisol is widely used as an indicator of stress in animal studies (Kellar *et al.*, 2015; Hunt *et al.*, 2017a; Trumble *et al.*, 2018), partially as a result of the rapid nature of the hypothalamic–pituitary–adrenocortical system’s response during stressful events. Progesterone, a key pregnancy hormone, is often used as an indicator of reproductive events such as age at sexual maturity, oestrous and pregnancy in a variety of mammalian species (Rolland *et al.*, 2005; Kellar *et al.*, 2006; Clark *et al.*, 2016; Hunt *et al.*, 2016; Robeck *et al.*, 2017; Pallin *et al.*, 2018).

In this study, extremely rare paired right and left earplugs from four individual baleen whales were examined to determine if individual lamina exhibit similar cortisol and progesterone concentrations in corresponding lamina and therefore interchangeable for endocrinology research. Determining if differences exist between corresponding baleen whale earplug lamina allows for improved analysis and interpretation of years to lifetime duration longitudinal studies, including establishing life events and providing insights into causal mechanisms and processes. Further, individual-level longitudinal data on biological and behavioural data are becoming increasingly available from a variety of biological matrices. Thus, two data transformation methods were evaluated in their utility to merge data by age or calendar year from multiple individuals: baseline correction and *Z*-score normalization. To date, no studies have investigated the potential hormone concentration differences within these corresponding sets of right and left earplugs.

## Methods

Corresponding sets of right and left earplugs from four baleen whales were used in this study: two individual baleen whales (#1120 and 1121) of unknown species and archived at the Smithsonian Museum of Natural History and two earplugs from recent strandings, one fin whale (*Balaenoptera physalus*, ID #1019) and one humpback whale (*Megaptera novaeangliae*, ID #1025, Table 1). Because of the inability to distinguish between ‘right’ and ‘left’ earplugs, designations were assigned at random for each baleen whale.

**Table 1:** Summary of whale earplugs used for this study

					Cortisol			Progesterone		
Whale ID	Species	Earplug	Estimated age (years)	Lamina *N*	Mean concentration (pg/g)	Baseline-corrected mean (%)	Mean *Z*-score	Mean concentration (pg/g)	Baseline-corrected mean (%)	Mean *Z*-score
1019	Fin whale	Right	1.5	3	2730 ± 340	29.2 ± 15.9	0.0± 1.0	680 ± 180	41.9 ± 21.0	0.0 ± 1.0
		Left	1.5	3	2530 ± 570	30.7 ± 29.5	0.0 ± 1.0	390 ± 60	25.7 ± 13.2	0.0 ± 1.0
1025	Humpback whale	Right	1.5	3	2740 ± 180	13.5 ± 7.3	0.0 ± 1.0	590 ± 10	1.6± 0.9	0.0 ± 1.0
		Left	1.5	3	1980 ± 100	8.0 ± 5.7	0.0 ± 1.0	610 ± 50	11.7 ± 6.5	0.0 ± 1.0
1120	Unknown	Right	15	30	1700 ± 60	13.3 ± 2.7	0.0 ± 1.0	790 ± 70	34.4 ± 4.9	0.0 ± 1.0
		Left	16	32	1410 ± 40	13.2 ± 2.4	0.0 ± 1.0	1000 ± 30	18.9 ± 2.3	−0.1 ± 1.0
1121	Unknown	Right	14	28	1900 ± 30	9.8 ± 1.7	0.0 ± 1.0	1420 ± 140	31.2 ± 5.0	0.0 ± 1.0
		Left	16	32	1750 ± 50	9.4 ± 2.2	−0.1 ± 0.8	1440 ± 30	11.8 ± 1.9	0.0 ± 1.1

Means all refer to the mean lamina hormone concentration over the entirety of the earplug. For both cortisol and progesterone, mean concentrations and standard deviation used for *Z*-score normalization and the baseline used for baseline correction for each whale for the right and left earplugs are provided. Baselines are calculated by averaging the three lowest hormone concentrations, except in the case of juvenile whales ID# 1019 and 1025, where only the lowest hormone concentration was used

### Aging and delamination

Aging and delamination methods for baleen whale earplugs have been described previously (Trumble *et al.*, 2018). Briefly, whales were aged to the nearest year by counting individual earplug layers (lamina), assuming a dark and light lamina equates to 1 year (growth layer groups, Gabriele *et al.*, 2010). After aging, laminae were separated, weighed (± 0.001 g), homogenized and stored at 4°C in nitrogen filled amber vials until lipid extraction. Lipids were extracted from individual earplug lamina using a Soxtec 2043 extraction system (FOSS) with 2:1 chloroform to methanol for 60 min at 160°C. After extraction, extracts were dried under nitrogen and stored at −80°C until analysis. Using enzyme-linked immunoassays (ELISA; Enzo Life Sciences, cortisol: ADI-901-071, progesterone: ADI-901011), extracted lipid aliquots were analysed for cortisol and progesterone in duplicate. Optical density values (Beckman Coulter DTX 880 Multimode Detector) were converted to hormone concentration in pg/g lipid. Cortisol in baleen whale earplugs has been assessed for linearity and accuracy (Trumble *et al.*, 2013). Pooled samples from three male and three female fin whales were serially diluted, and the resultant values were compared to the ELISA kit progesterone standard to ascertain the binding affinity of progesterone to the assay antibodies (Hunt *et al.*, 2017b). Pooled samples from three male and three female fin whales were then combined and spiked with known serial dilution of the standards to compare observed concentration to known standard concentration to determine accuracy of the assay for progesterone (Grotjan and Keel, 1996; Hunt *et al.*, 2017b).

### Absolute hormone values in right and left earplugs

Absolute hormone concentrations (pg/g lipid) for corresponding right and left earplug laminae were compared using a mixed-model framework as a regression analysis to account for non-independence of data, with individual whale ID as the random effect (Bates *et al.*, 2015; Bartón, 2019): lmer (left earplug absolute hormone ~ right earplug absolute hormone + 1|WhaleID, data = data), where cortisol concentrations from right and left earplugs were compared and the progesterone concentrations from right and left earplugs were compared. Conditional *r*^2^ is reported using MuMIn R package for the variance explained by fixed and random effects (Nakagawa and Schielzeth, 2012; Bartón, 2019).

Furthermore, the difference between corresponding lamina absolute hormone concentration in right and left earplugs was calculated, from which a 95% confidence interval (CI) was derived (Table 2). For example, the hormone concentration of lamina A in the right earplug is subtracted from lamina A in the left earplug and recorded as the difference between the two laminae. This is repeated for all laminae in the right and left earplugs of an individual; these differences are then used to calculate the 95% CI around the mean of the differences. If the 95% CI of the differences between the right and left earplug includes zero, this indicates that the hormones in corresponding lamina of the right and left earplug are no different from zero.

### Controlling for differing lifetime mean hormone concentration in individual subjects

Mean lifetime hormone concentration was compared between corresponding earplugs, in absolute (pg/g lipid), baseline-corrected (%) and *Z*-score-normalized values (Table 1). Mean lifetime hormone values were calculated as the mean hormone value per lamina for each right and left earplug (sum of hormone values divided by total number of laminae for each earplug). For each earplug, hormone concentrations were baseline-corrected and *Z*-score-normalized (i.e. calculated independently for each right and left earplug from each individual). To calculate baseline-corrected hormone values, ‘baseline’ levels were defined as the mean of the three lowest hormone values for each earplug (Table 1, Trumble *et al.*, 2018). Due to the age of the juvenile whales (<2 years), baselines for ID# 1019 and 1025 were assigned as the single lowest hormone concentration for each individual earplug (Table 1). Baseline-corrected values, as percent change from the baseline for each lamina, were calculated as: (lamina hormone concentration−baseline)/baseline)*100%, which is derived from Trumble *et al.,* (2018). *Z*-scores for each lamina in a whale’s earplug were calculated as ((lamina absolute hormone concentration−mean of earplug lamina)/standard deviation of earplug lamina). These resulting data have a mean of zero and are proportional to the standard deviation. A *Z*-score of one for cortisol in a single baleen whale earplug lamina would indicate that over 6 months this individual produced elevated cortisol one standard deviation above its mean cortisol production over its life.

**Figure 1:**
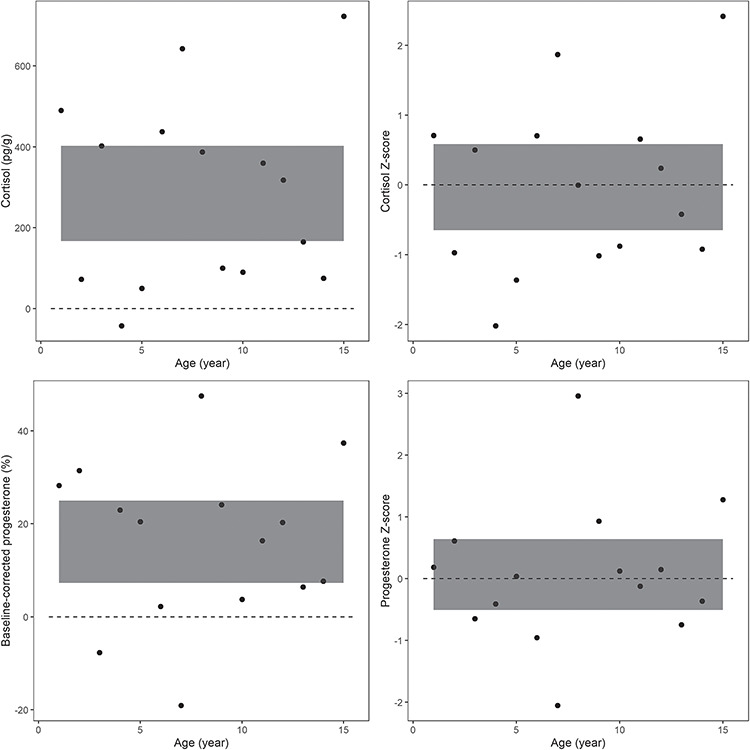
The absolute, baseline-corrected and *Z*-score-hormone differences between the right and left earplugs of baleen whale ID# 1120. **A.** Absolute cortisol hormone concentration and **C.** baseline-corrected progesterone did not always include zero in the 95% CI when comparing the differences between corresponding sets of earplugs (shaded areas represent 95% CI of differences between right and left earplug laminae, 167–402 pg/g and 7.3–25%, respectively). However, **B.** cortisol *Z*-score and **D.** progesterone *Z*-score always exhibited a 95% CI that included zero for all earplugs (shaded areas represent 95% CI of differences between *Z*-scores of right and left earplug laminae, −0.6–0.6 and −0.5–0.6, respectively)

To assess differences in mean lifetime hormone concentrations, a Wilcoxon signed-rank test was used due to the non-normal distribution of the differences between the pairs of corresponding laminae. Baseline-corrected and *Z*-score-normalized hormones were used to control for differing lifetime mean hormone concentration in individual earplugs. Specifically, the difference between each layer of corresponding sets of individual’s earplugs was calculated for baseline-corrected and *Z*-score-normalized hormones, from which a 95% CI from the mean was calculated for each individual (Table 2, Fig. 1).

**Table 2:** 95% confidence interval (CI) of differences in absolute, baseline-corrected and *Z*-score-normalized hormones in corresponding lamina between right and left earplugs for both cortisol and progesterone

	95% CI cortisol			95% CI Progesterone		
Whale ID	Absolute	Baseline-corrected	*Z*-score	Absolute	Baseline-corrected	Z-score
1019	−927–519	−36.8–39.9	−0.7–0.7	−729–160	−76.7–44.4	−2.0–2.0
1025	−1090–428^*^	−19.9–9.1	−1.3–1.3	−81–106	−3.7–24.1	−2.0–1.9
1120	167–402^*^	−6.9–6.1	−0.6–0.6	−326–−86^*^	7.3–25^*^	−0.5–0.6
1121	132–284^*^	−1.1–6.6	−0.2–0.8	−315–254	7.7–29.5^*^	−0.8–0.6

^*^ indicates when the 95% CI did not include zero

## Results

### Assays

Baleen whale earplug cortisol and progesterone intra-assay coefficient of variation (CV) for all ELISAs were 7.7 ± 6.6 and 6.6 ± 5.5%, respectively (mean ± standard deviation). Progesterone assay parallelism was exhibited for both female and male fin whale pooled samples (*N* = 6, ANCOVA, *F* = 0.129, *P* > 0.05 for females and *F* = 1.8, *P* > 0.05 for males, Fig. S1). Progesterone assay accuracy exhibited a positive linear relationship of observed versus expected concentration (slope = 1.3, *r*^2^ = 0.99). Cortisol assay validation has been shown previously for baleen whale earplugs (Trumble *et al.*, 2013).

### Absolute hormone values in right and left earplugs

Absolute cortisol, as well as progesterone concentrations compared between corresponding lamina in the right and left earplugs, had a positive significant relationship (cortisol: conditional *r*^2^ = 0.88, *t*_1, 3.0_ = 9.79, *P* = 0.002, progesterone: conditional *r*^2^ = 0.93, *t*_1, 3.0_ = 3.6, *P* = 0.03, Fig. S2).

### Controlling for differing lifetime mean hormone concentration in individual subjects

Cortisol and progesterone were both baseline-corrected and *Z*-score-normalized to determine mean lifetime hormones. The 95% CI for baseline-corrected cortisol always included zero while baseline-corrected progesterone did not include zero for whale ID# 1120 and 1121. *Z*-score normalization for both cortisol and progesterone always included zero (Table 2, Fig. 1).

The lifetime mean lamina hormone value for each individual’s right and left earplug was compared (Table 1). Mean absolute cortisol concentrations for the right and left earplugs for two individuals were significantly different (ID# 1120 and 1121, Wilcoxon signed-rank test, *P* = 0.0001 and *P* = 0.001, respectively), whereas mean baseline-corrected and *Z*-score normalized cortisol did not differ (Fig. S3, Wilcoxon signed-rank test, *P* > 0.2). Mean absolute progesterone was significantly different for whale ID# 1120 (Wilcoxon signed-rank test, *P* = 0.008), and baseline-corrected progesterone was significantly different for both whale ID# 1120 and 1121 (Wilcoxon signed-rank test, *P* = 0.004, *P* = 0.01 respectively), whereas mean *Z*-score-normalized progesterone values were no different (Table 1, Fig. S4, Wilcoxon signed-rank test, *P* > 0.5).

## Discussion

Results from this study demonstrate significant positive relationships in absolute cortisol and progesterone concentrations in corresponding lamina between earplugs within individual whales. This supports the use of either the right or left earplug for endocrinology research, which is valuable since museums may only have one earplug from an individual, or deceased, stranded individuals may be positioned in such a way that only one earplug is accessible. However, of the two data transformation techniques analysed, only *Z*-score-normalized hormones always included zero when examining the 95% confidence interval around the mean between the right and left earplugs. Furthermore, *Z*-score normalization provided an identical lifetime mean hormone value from which to compare across individuals (Fanson *et al.*, 2017). This allows researchers to use *Z*-score-normalized hormones to merge individual hormone trends. Therefore, we conclude that *Z*-score transformation adequately corrects for variance between earplugs, appropriate for use in studying stress and reproduction by age and calendar year (Trumble *et al.*, 2018). *Z*-score normalization does remove individual variability from hormone data, where individuals with significantly higher lifetime hormone means would be combined with individuals with significantly lower lifetime hormone means. This should be taken into consideration when making the decision to *Z*-score normalize hormone data. These findings indicate right and left earplugs can be used interchangeably after *Z*-score normalization to reconstruct hormone profiles to examine long-term trends in baleen whales.

### Assays

Parallelism and accuracy require a great deal of sample mass from extremely rare samples and therefore could not be assessed for all species (Hunt *et al.*, 2017b). Due to insufficient sample mass per earplug, aliquots for parallelism and accuracy tests were unavailable for all species; however, given the validations performed for progesterone and cortisol during this and a previous study, we assume these validations for earplug hormones extend to all Mysticetes (Trumble *et al.*, 2013; Hunt *et al.*, 2017b).

### Aging and delamination

Assuming formation of lamina within earplugs is biannual (Gabriele *et al.*, 2010), difficulties arise when aging earplugs from older individuals. (Purves, 1955; Lockyer, 1972). As the earwax accrues, the lamina becomes increasingly compacted as age and size of the earplug advances (Purves, 1955; Lockyer, 1972). Furthermore, many species of cetaceans, including baleen whales, show behavioural and physiological laterality and asymmetry, which may indicate that external ear canals are slightly different shapes and sizes (Galatius and Jespersen, 2005; MacLeod *et al.*, 2007; Canning *et al.*, 2011; Pyenson *et al.*, 2012). Therefore, compaction could influence earplug and lamina shape and distinction, leading to variability in delamination, aging and ultimately longitudinal comparisons. However, while errors in aging may lead to an offset in the time between right and left earplugs (Fig. S3, Fig. S4), overall lifetime trends remain consistent. Errors associated with aging and delamination described in this study did not introduce data bias, though techniques for earplug delamination continue to be improved to reduce any possible differences.

**Figure 2:**
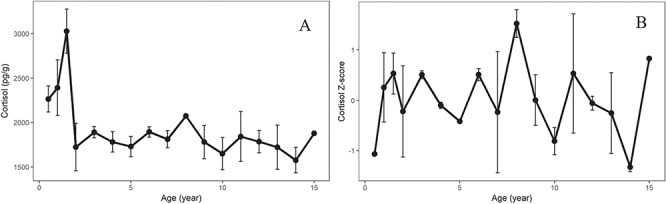
Here, we demonstrate the differences that can arise when assessing hormone trends across multiple individuals when using absolute cortisol concentration as compared to *Z*-score-normalized cortisol. **A**. All whales combined by age for absolute cortisol concentration and **B**. all whales combined by age for cortisol *Z*-score. Vertical bars represent standard error. Any points with no visible error bars are present, but very small

### Absolute hormone values in right and left earplugs

Previous studies have shown similarities in hormone concentrations between different tissues from within individuals (Kellar *et al.*, 2013; Charapata *et al.*, 2018; Mingramm *et al.*, 2019). In addition, a few studies have investigated hormone similarity from the same tissue in different locations within the body (Kellar *et al.*, 2006; Cattet *et al.*, 2017; Mello *et al.*, 2017; Charapata *et al.*, 2018). Kellar *et al*. (2006) showed that blubber depth had no effect on progesterone concentration in a northern right whale dolphin (*Lissodelphis borealis*) while blubber sampled at different locations in the body were significantly different, though the differences were relatively small. Mello *et al*. (2017) reported different blubber sampling locations in humpback whales (*Megaptera novaeangliae*) which resulted in similar hormone concentrations, except for testosterone from the dorsal fin. Charapata *et al.* (2018) showed extensive variability in individual walrus (*Odobenus rosmarus*) bone concentrations but showed that hormones extracted from cortical bone across the walrus skeleton were similar. Hair from brown bears (*Ursus arctos*) also revealed similar hormone concentrations in hair taken from different locations on the individual’s pelt (Cattet *et al.*, 2017). The results of the mixed model from the present study demonstrate that as cortisol or progesterone concentration increases in the left earplug, they increase in the right earplug. These similar trends in hormone data (i.e. between right and left earplugs) indicate a consistent excretion pathway for hormone deposition into the developing earplug. Therefore, we surmise that baleen whale earplugs are suitable in assessing longitudinal endocrinological trends in Mysticetes.

### Utility of *Z*-score-normalizing longitudinal hormone values

The concept of hormone ‘baselines’ is not straightforward across and within disciplines (Bortolotti *et al.*, 2008; Houser *et al.*, 2011; Fair *et al.*, 2014; Rolland *et al.*, 2017). Colloquially, a baseline in animal studies is a minimum or fixed reference point which can be used in comparing between or among morphological, behavioural or physiological data. Many studies involving avian species define a baseline value as the measured concentration of a particular analyte during an unstressed period, usually taken immediately after capture (Bortolotti *et al.*, 2008). Similarly, studies in free-ranging bottlenose dolphins, *Tursiops truncatus*, establish baseline analyte values after capture (Fair *et al.*, 2014), whereas studies in managed dolphins use baseline values from voluntary blood draws (Houser *et al.*, 2011). Marine mammal research involving free-ranging animals must rely on innovative techniques or opportune sampling to determine baselines. For example, Rolland *et al.* (2017) defined baseline glucocorticoid concentration for North Atlantic right whales as the mean faecal glucocorticoid concentration of healthy individuals. These techniques for assessing hormone baselines are designed for the time scales over which change in hormone concentration is being examined—minutes to hours in most cases.

Here, we suggest calculating a lifetime hormone baseline by averaging the hormone values of each lamina per earplug for one consistent value across the timeline, which, when transformed, would be a *Z*-score of zero (Table 1). When comparing the corresponding lamina from the right and left earplugs within the same individual, only baseline-corrected cortisol and *Z*-score-normalized cortisol and progesterone values resulted in similar lifetime hormone means, where the 95% CI of the mean of the differences included zero. If zero is included in the 95% CI for the mean of the differences, this indicates the right and left earplug hormone values per lamina are similar. However, if zero is not included in the 95% CI for the mean of the differences, then right and left earplug hormone values per lamina are different. Hormone values from juvenile whales (ID# 1019, 1025) do create wider 95% CIs than for the older animals (ID# 1120, 1121), though these confidence intervals still include zero. The right and left earplugs for ID# 1120 and 1121, aged and delaminated differently, could explain why absolute hormone concentration 95% CI did not include zero. Therefore, baseline correction and *Z*-score normalization were used to control for both natural hormone variability (Fanson *et al.*, 2017; Charapata *et al.*, 2018; Trumble *et al.*, 2018) and for variation in aging and delamination of lamina to merge and examine trends of multiple individuals by age or calendar year.

Baleen whale earplugs for which individual lamina have been *Z*-score-normalized (Table 1): (i) provide a means with which to compare changes in hormone trends across individuals, populations (Clark *et al.*, 2017; Trumble *et al.*, 2018) or species, (ii) creates an equal lifetime hormone mean from which to compare across individuals with natural variability in hormone expression over their lifetimes (Jenkins *et al.*, 2014; Charapata *et al.*, 2018; Trumble *et al.*, 2018) and (iii) standardizes the variance, so that one animal does not overly influence the trends (Fanson *et al.*, 2017). We acknowledge that using *Z*-scores reduces individual variability in hormone expression as well as difference between individuals. Therefore, the researcher must be mindful about its limitations, particularly when interpreting transformed data.

### Combining hormone profiles from multiple individuals

To illustrate the utility of using a method of calculating a lifetime hormone baseline that allows comparison among individuals, mean cortisol concentrations (pg/g lipid) as well as *Z*-scores were compared across the four individuals in this study as a function of age (Fig. 2A and B). These graphs demonstrate that interpretation of results may differ with regards to using absolute hormone concentrations or *Z*-score-normalized hormone values. As described here, absolute cortisol values plotted over 15 years reveal 6-month-old calves have higher cortisol *Z*-scores than later in life and a peak at 1.5 years of age (Fig. 2A). However, while *Z*-score normalization reveals a similar cortisol increase at 1.5 years of age, corresponding to weaning age (Chittleborough, 1958; Lockyer, 1984), 6-month old calves have lower cortisol *Z*-scores with a peak in cortisol occurring at 6–9 years of age (Fig. 2B). Previous studies have suggested that this peak between 6 and 9 may be associated with onset of sexual maturity (Trumble *et al.*, 2013). There is a notable difference of interpretation when comparing *Z*-score-normalized data across lifetimes of multiple whales as compared to using absolute cortisol concentrations in the same manner. Individual lifetime hormone baseline variability between different individuals must be considered when assessing trends across multiple individuals (Fig. 2, Trumble *et al.*, 2018).

## Conclusion

In this study, we have shown that hormones in the left and right earplugs of baleen whales exhibit similar trends and are therefore interchangeable. In addition, we have demonstrated that hormones must be *Z*-score-normalized to merge data and analyse decades-long trends. Analysing the effectiveness of baseline-correcting and *Z*-score-normalizing hormones across an individual’s lifetime is vital for researchers using continuously growing biological matrices with the intention of comparison across individuals.

## Supplementary material

Supplementary material is available at *Conservation Physiology* online.

## Funding

This work was supported by a grant from the Office of Naval Research (N00014-17-S-B001) to S.J.T. and S.U.

## Conflict of interest statement

The authors declare that the research was conducted in the absence of any commercial or financial relationships that could be construed as a potential conflict of interest.

## Supplementary Material

suppl_data_coaa055

## References

[ref1] Bartón K (2019) MuMIn: Multi-Model Inference. R package version 1: 15 https://CRAN.R-project.org/package=MuMIn.

[ref2] Bates D, Mächler M, Bolker B, Walker S (2015) Fitting linear mixed-effects models using lme4. J. Statistical Software 67: 1–48.

[ref3] Bortolotti GR, Marchant TA, Blas J, German T (2008) Corticosterone in feathers is a long-term, integrated measure of avian stress physiology. Funct Ecol 22: 494–500.

[ref4] Canning C, Crain D, Eaton TS Jr, Nuessly K, Friedlaender A, Hurst T, Parks S, Ware C, Wiley D, Weinrich M (2011) Population-level lateralized feeding behaviour in North Atlantic humpback whales, *Megaptera novaeangliae*. Anim Behav 82: 901–909.

[ref5] Cattet M, Stenhouse GB, Janz DM, Kapronczai L, Anne Erlenbach J, Jansen HT, Nelson OL, Robbins CT, Boulanger J (2017) The quantification of reproductive hormones in the hair of captive adult brown bears and their application as indicators of sex and reproductive state. Conserv Physiol 5: 1–19. doi: 10.1093/conphys/cox032. PMC5452076 28580147

[ref6] Charapata P, Horstmann L, Jannasch A, Misarti N (2018) A novel method to measure steroid hormone concentrations in walrus bone from archaeological, historical, and modern time periods using liquid chromatography/tandem mass spectrometry. Rapid Commun Mass Spectrom 32: 1999–2023. 30192037 10.1002/rcm.8272 PMC6282614

[ref7] Chittleborough R (1958) The breeding cycle of the female humpback whale, *Megaptera nodosa* (Bonnaterre). Mar Freshw Res 9: 1.

[ref8] Clark CT, Fleming AH, Calambokidis J, Kellar NM, Allen CD, Catelani KN, Robbins M, Beaulieu NE, Steel D, Harvey JT (2016) Heavy with child? Pregnancy status and stable isotope ratios as determined from biopsies of humpback whales. Conserv Physiol 4: 1–13. 27766149 10.1093/conphys/cow050 PMC5070529

[ref9] Clark CT, Horstmann L, Misarti N (2017) Quantifying variability in stable carbon and nitrogen isotope ratios within the skeletons of marine mammals of the suborder Caniformia. J Archaeol Sci Rep 15: 393–400.

[ref10] Fair PA, Schaefer AM, Romano TA, Bossart GD, Lamb SV, Reif JS (2014) Stress response of wild bottlenose dolphins (*Tursiops truncatus*) during capture–release health assessment studies. Gen Comp Endocrinol 206: 203–212. 25019655 10.1016/j.ygcen.2014.07.002

[ref11] Fanson KV et al. (2017) One size does not fit all: monitoring faecal glucocorticoid metabolites in marsupials. Gen Comp Endocrinol 244: 146–156. 26478011 10.1016/j.ygcen.2015.10.011

[ref12] Gabriele CM, Lockyer C, Straley JM, Jurasz CM, Kato H (2010) Sighting history of a naturally marked humpback whale (*Megaptera novaeangliae*) suggests ear plug growth layer groups are deposited annually. Mar Mam Sci 26: 443–450.

[ref13] Galatius A, Ås J (2005) Bilateral directional asymmetry of the appendicular skeleton of the harbor porpoise (*Phocoena phocoena*). Mar Mam Sci 21: 401–410.

[ref14] Grotjan H, Keel B (1996) Data interpretation and quality control In EP Diamandis, TK Christopoulos, eds. Immunoassay. Academic Press San Diego, pp 51–93.

[ref15] Houser DS, Yeates LC, Crocker DE (2011) Cold stress induces an adrenocortical response in bottlenose dolphins (*Tursiops truncatus*). J Zoo Wildlife Med 42: 565–571. 10.1638/2010-0121.1 22204049

[ref16] Hunt KE, Lysiak NS, Moore M, Rolland RM (2017a) Multi-year longitudinal profiles of cortisol and corticosterone recovered from baleen of North Atlantic right whales (*Eubalaena glacialis*). Gen Comp Endocrinol 254: 50–59. 28919447 10.1016/j.ygcen.2017.09.009

[ref17] Hunt KE, Lysiak NS, Moore MJ, Rolland RM (2016) Longitudinal progesterone profiles in baleen from female North Atlantic right whales (Eubalaena glacialis) match known calving history. Conserv Physiol 4: 1–9. cow014. 27293762 10.1093/conphys/cow014 PMC4864594

[ref18] Hunt KE, Lysiak NS, Robbins J, Moore MJ, Seton RE, Torres L, Buck CL (2017b) Multiple steroid and thyroid hormones detected in baleen from eight whale species. Conserv Physiol 5: 1–14. doi: 10.1093/conphys/cox061. PMC5691779 29230292

[ref19] Hunt KE, Stimmelmayr R, George C, Hanns C, Suydam R, Brower H, Rolland RM (2014) Baleen hormones: a novel tool for retrospective assessment of stress and reproduction in bowhead whales (*Balaena mysticetus*). Conserv Physiol 2: 1–12. cou030–cou030. 10.1093/conphys/cou030 PMC4806734 27293651

[ref20] Jenkins BR, Vitousek MN, Hubbard JK, Safran RJ (2014) An experimental analysis of the heritability of variation in glucocorticoid concentrations in a wild avian population. Proc R Soc B 281: 20141302. 10.1098/rspb.2014.1302 PMC4123711 25056627

[ref21] Karpovich SA, Skinner JP, Kapronczai LA, Smith JA, Janz DM (2018) Examination of relationships between stable isotopes and cortisol concentrations along the length of phocid whiskers. Mar Mam Sci 35: 395–415. doi:10.1111/mms.12546

[ref22] Kellar N, Keliher J, Trego M, Catelani K, Hanns C, George J, Rosa C (2013) Variation of bowhead whale progesterone concentrations across demographic groups and sample matrices. Endanger Species Res 22: 61–72.

[ref23] Kellar NM, Catelani KN, Robbins MN, Trego ML, Allen CD, Danil K, Chivers SJ (2015) Blubber cortisol: a potential tool for assessing stress response in free-ranging dolphins without effects due to sampling. PLoS One 10: 1–16. e0115257. 10.1371/journal.pone.0115257 PMC4314064 25643144

[ref24] Kellar NM, Trego ML, Marks CI, Dizon AE (2006) Determining pregnancy from blubber in three species of delphinids. Mar Mam Sci 22: 1–16.

[ref25] Landys MM, Ramenofsky M, Wingfield JC (2006) Actions of glucocorticoids at a seasonal baseline as compared to stress-related levels in the regulation of periodic life processes. Gen Comp Endocrinol 148: 132–149. 16624311 10.1016/j.ygcen.2006.02.013

[ref26] Lockyer C (1972) The age at sexual maturity of the southern fin whale (*Balaenoptera physalus*) using annual layer counts in the ear plug. ICES J Mar Sci 34: 276–294.

[ref27] Lockyer C (1984) Review of baleen whale (Mysticeti) reproduction and implications for management. Reports of the International Whaling Commission, Special Issue 6: 27–50.

[ref28] Lysiak NSJ, Trumble SJ, Knowlton AR, Moore MJ (2018) Characterizing the duration and severity of fishing gear entanglement on a North Atlantic right whale (*Eubalaena glacialis*) using stable isotopes, steroid and thyroid hormones in baleen. Front Mar Sci 5: 1–13. doi: 10.3389/fmars.2018.00168.29552559

[ref29] MacLeod CD, Reidenberg JS, Weller M, Santos MB, Herman J, Goold J, Pierce GJ (2007) Breaking symmetry: the marine environment, prey size, and the evolution of asymmetry in cetacean skulls. The Anatomical Record: Advances in Integrative Anatomy and Evolutionary Biology: Advances in Integrative Anatomy and Evolutionary Biology 290: 539–545. 10.1002/ar.20539 17516443

[ref30] Mello D, Colosio A, Marcondes M, Viau P, Oliveira C (2017) Feasibility of using humpback whale blubber to measure sex hormones. J Exp Mar Biol Ecol 486: 32–41.

[ref31] Mingramm FMJ, Dunlop RA, Blyde D, Whitworth DJ, Keeley T (2019) Evaluation of respiratory vapour and blubber samples for use in endocrine assessments of bottlenose dolphins (*Tursiops* spp.). Gen Comp Endocr 274: 37–49. 30605661 10.1016/j.ygcen.2018.12.015

[ref32] Nakagawa S, Schielzeth H (2012) The mean strikes back: mean–variance relationships and heteroscedasticity. Trends Ecol Evol 27: 474–476. 22578987 10.1016/j.tree.2012.04.003

[ref33] Pallin L, Robbins J, Kellar N, Bérubé M, Friedlaender A (2018) Validation of a blubber-based endocrine pregnancy test for humpback whales. Conserv Physiol 6: coy031. doi: 10.1093/conphys/coy031. PMC6009693 29942518

[ref34] Purves PE (1955) The Wax Plug in the External Auditory Meatus of the Mysticeti. Discovery Rep. 27: 293–302.

[ref35] Pyenson ND, Goldbogen JA, Vogl AW, Szathmary G, Drake RL, Shadwick RE (2012) Discovery of a sensory organ that coordinates lunge feeding in rorqual whales. Nature 485: 498–501. 22622577 10.1038/nature11135

[ref36] Robeck TR, Steinman KJ, O’Brien JK (2017) Characterization and longitudinal monitoring of serum androgens and glucocorticoids during normal pregnancy in the killer whale (*Orcinus orca*). Gen Comp Endocrinol 247: 116–129. 28126344 10.1016/j.ygcen.2017.01.023

[ref37] Rolland RM, Hunt KE, Kraus SD, Wasser SK (2005) Assessing reproductive status of right whales (*Eubalaena glacialis*) using fecal hormone metabolites. Gen Comp Endocrinol 142: 308–317. 15935157 10.1016/j.ygcen.2005.02.002

[ref41] Rolland RM, Hunt KE, Kraus SD, Wasser SK, Wasser SK, Wasser SK (2017) Fecal glucocorticoids and anthropogenic injury and mortality in North Atlantic right whales Eubalaena glacialis. Endang Species Res 34: 417–429. 10.3354/esr00866

[ref39] Trumble SJ, Norman SA, Crain DD, Mansouri F, Winfield ZC, Sabin R, Potter CW, Gabriele CM, Usenko S (2018) Baleen whale cortisol levels reveal a physiological response to 20th century whaling. Nat Commun 9: 1–8. 30389921 10.1038/s41467-018-07044-w PMC6215000

[ref40] Trumble SJ, Robinson EM, Berman-Kowalewski M, Potter CW, Usenko S (2013) Blue whale earplug reveals lifetime contaminant exposure and hormone profiles. Proc Natl Acad Sci 110: 16922–16926. 24043814 10.1073/pnas.1311418110 PMC3801066

