# Supplementary material for: Hormone comparison between right and left baleen whale earplugs
Source: Conserv Physiol. 2020 Jun 24;8(1):coaa055. doi: 10.1093/conphys/coaa055 (PMC7311829; doi:10.1093/conphys/coaa055)
Supplement: suppl_data_coaa055 [file suppl_data_coaa055.zip › Crainetal_R_L_ConsPhysSupplementary_20200515.docx]

**Supplementary Information for “Hormone comparison between right and left baleen whale earplugs”**

Authors: Danielle D. Crain^1^, (254) 710-2559, ddiancrain@gmail.com

Amanda Thomas^1^, (254) 710-2559, Amanda_Thomas3@baylor.edu

Farzaneh Mansouri^2^, (254) 710-2303, Farzaneh_Mansouri@baylor.edu

Charles W. Potter^3^, (202) 633-1261, POTTERC@si.edu

Sascha Usenko^1,2,4^, (254) 710-2302, Sascha_Usenko@baylor.edu

Stephen J. Trumble^1*^, (254) 710-2128, Stephen_Trumble@baylor.edu

^1^Department of Biology, Baylor University, Waco TX, 76706, USA

^2^Department of Environmental Science, Baylor University, Waco TX 76706, USA

^3^Department of Vertebrate Zoology, Smithsonian Institution National Museum of Natural History, Wash DC 20013 USA

^4^Department of Chemistry and Biochemistry, Baylor University, Waco TX 76706, USA

*Corresponding author


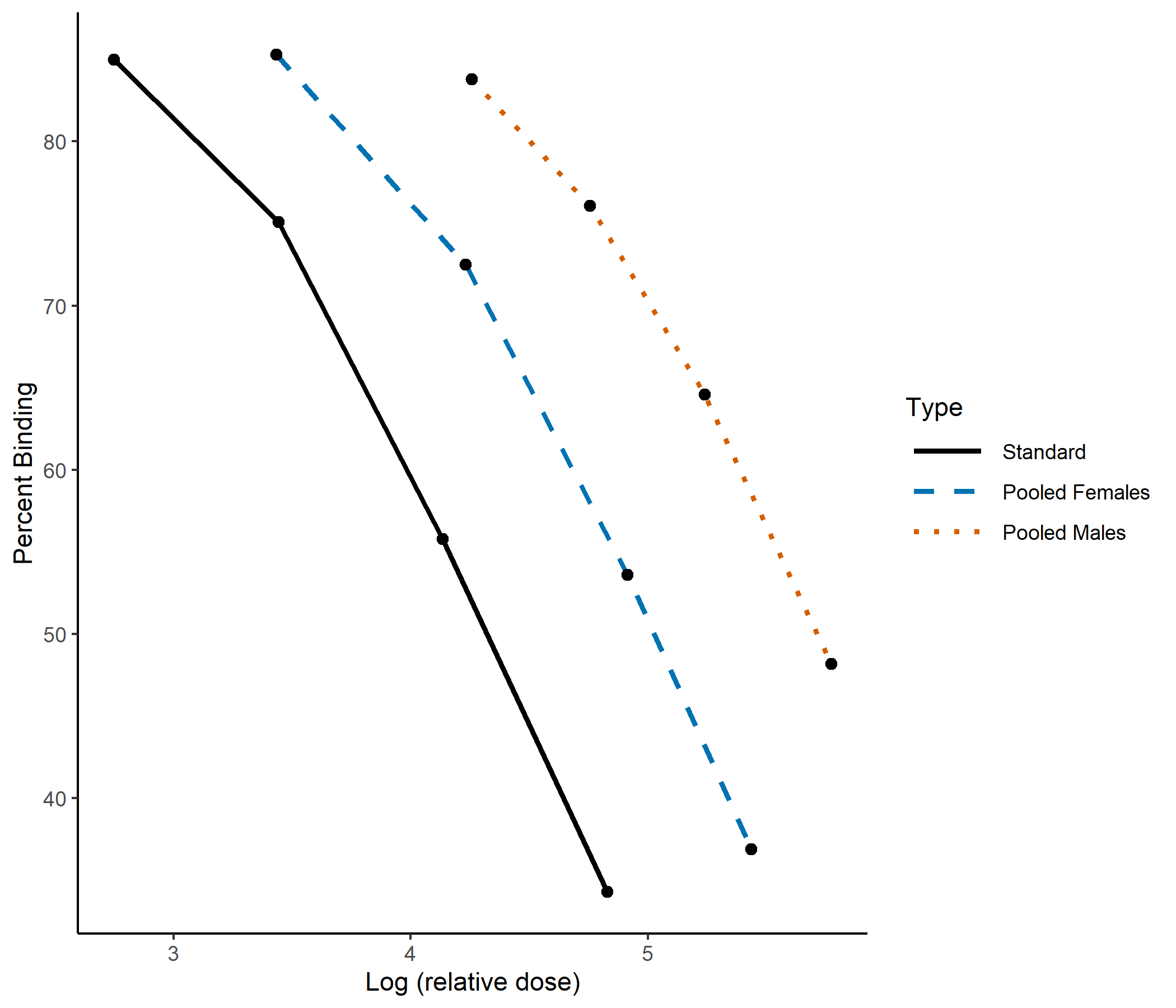


**Figure S1.** Progesterone parallelism, showing pooled female and male samples have slopes no different from the serially diluted standard concentrations (ANCOVA, F = 0.129, p > 0.05 for females and F = 1.8, p > 0.05 for males).


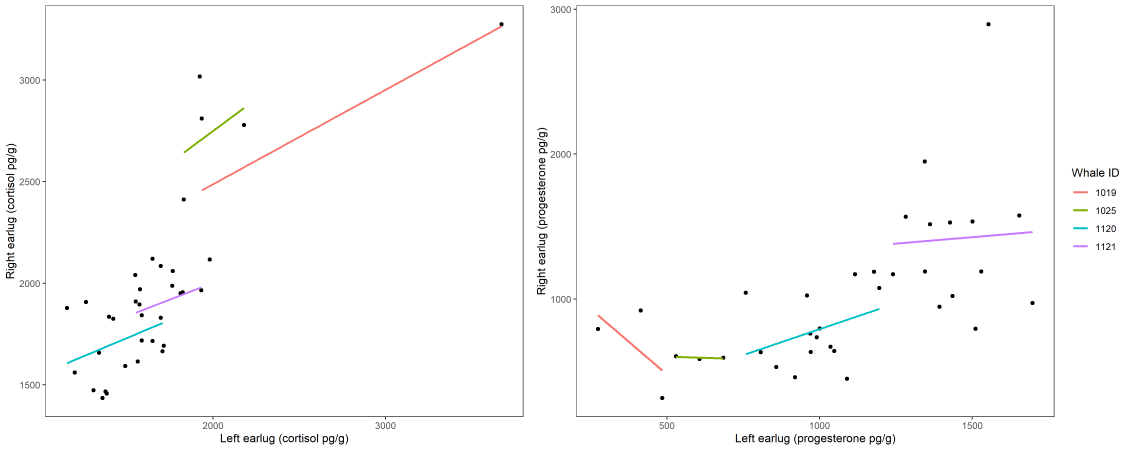


**Figure S2.** Regression of right earplug compared to left earplug for cortisol (left image) and progesterone (right image). Color of lines indicates whale ID.


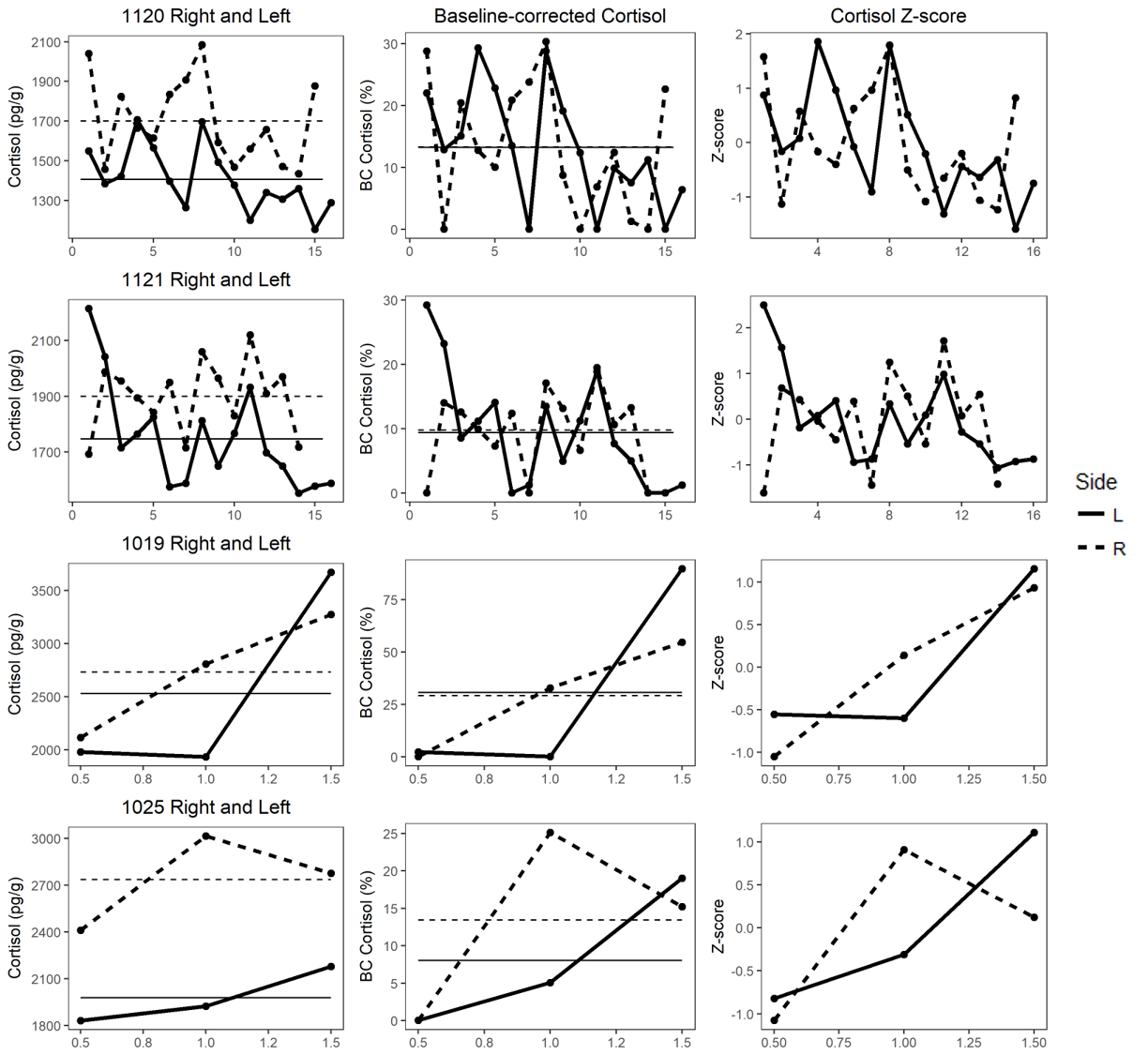


**Figure S3.** Absolute cortisol concentrations (left column), baseline-corrected cortisol (middle column), and Z-scores (right column) for all baleen whales in this study. Horizontal lines indicate the averages for each right and left individual earplug from each whale. Right earplugs happened to be higher in this study for cortisol, “right” and “left” assignations were assigned at random. Solid is left and dashed is right. The Z-score data does not have these horizontal lines because Z-scores are normalized to the average or zero (Table 1).


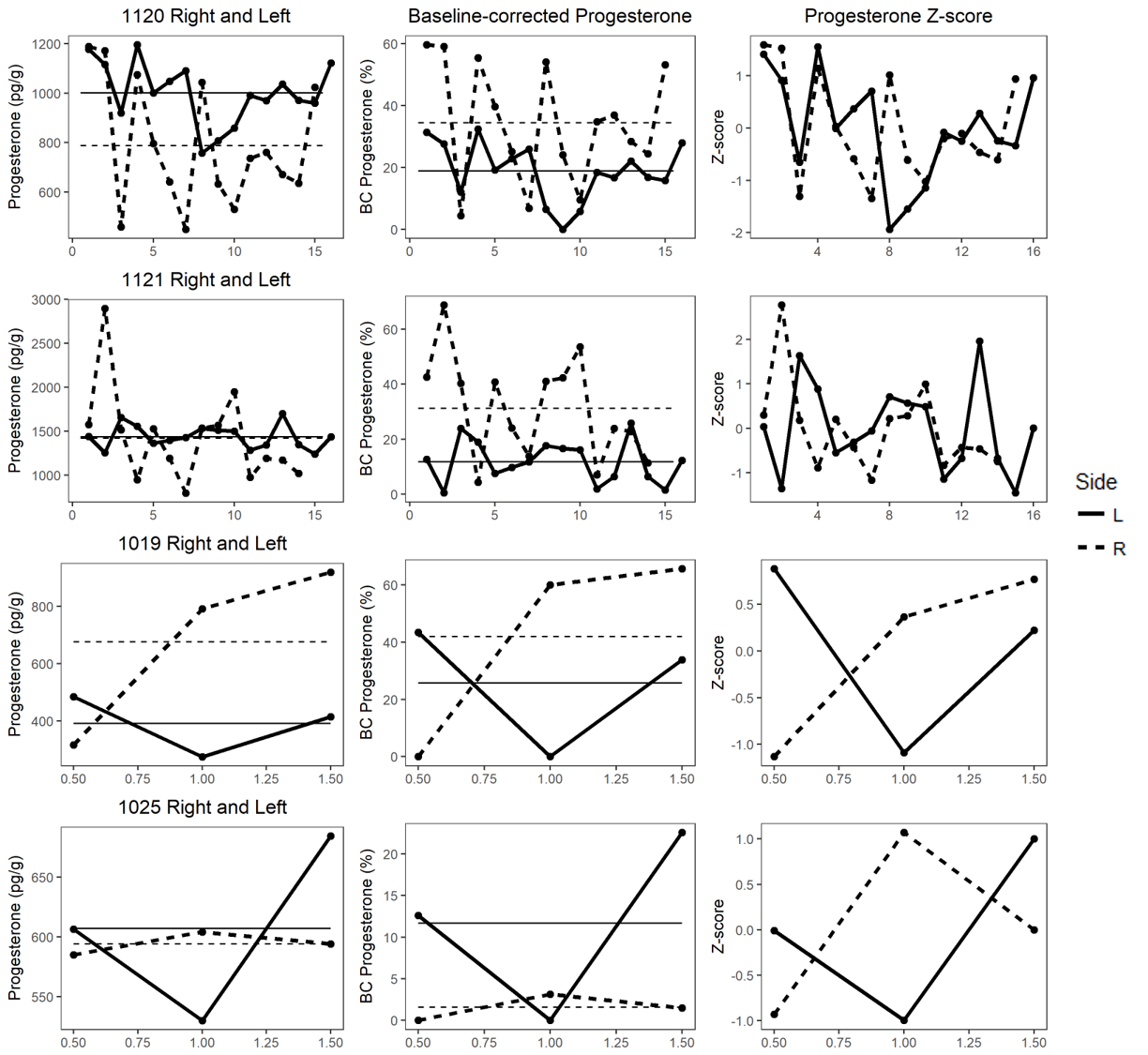


**Figure S4.** Absolute progesterone concentrations (left column), baseline-corrected progesterone (middle column), and Z-scores (right column) for all baleen whales in this study. Horizontal lines indicate the averages for each right and left individual earplug from each whale. Solid is left and dashed is right. The Z-score data does not have these horizontal lines because Z-scores are normalized to the average, which would be a Z-score of zero (Table 1).
