# Supplementary figures and images for: Hormone comparison between right and left baleen whale earplugs
Source: Conserv Physiol. 2020 Jun 24;8(1):coaa055. doi: 10.1093/conphys/coaa055 (PMC7311829; doi:10.1093/conphys/coaa055)

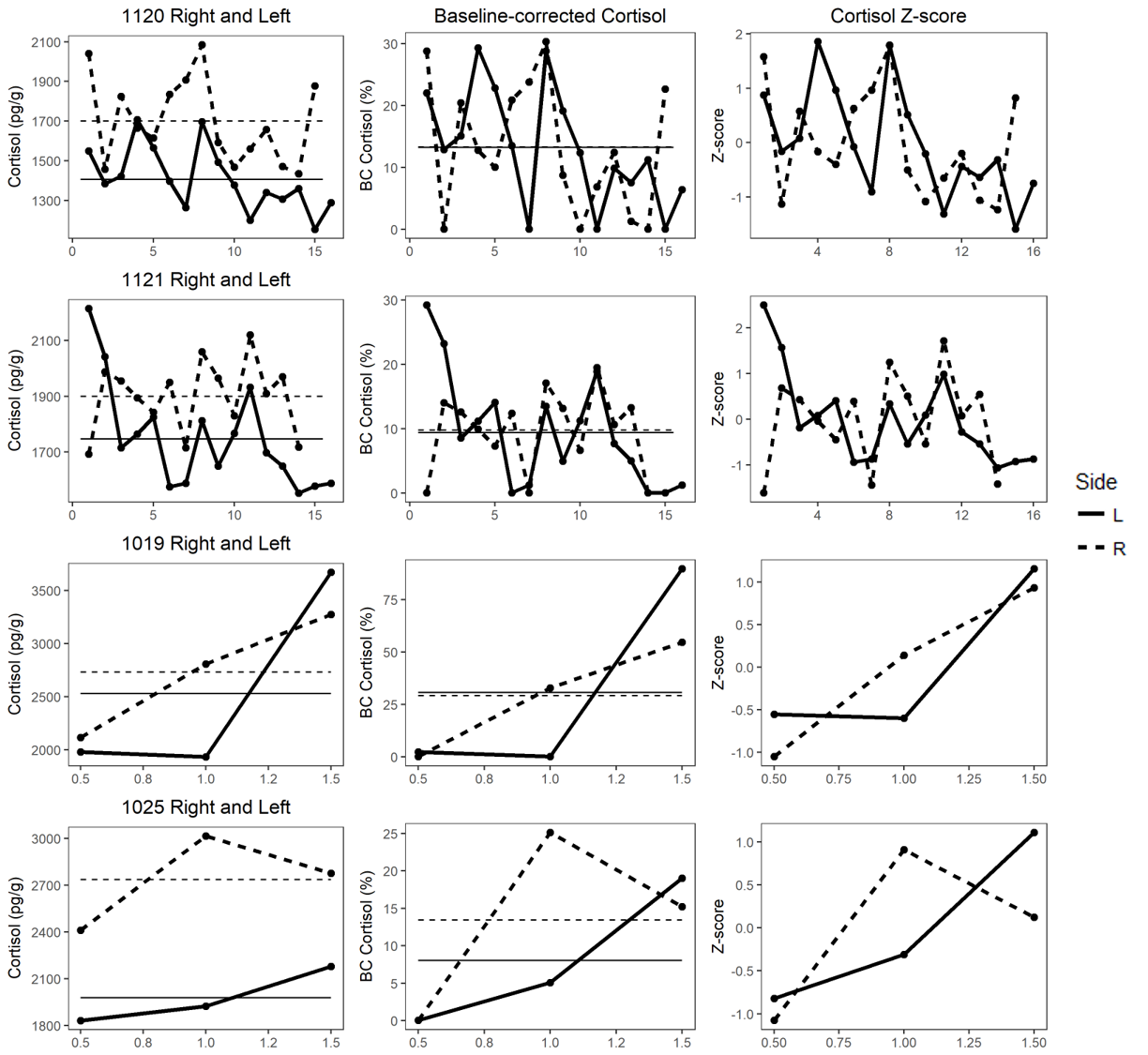

Supplement: suppl_data_coaa055 [file suppl_data_coaa055.zip › FigS3.png]

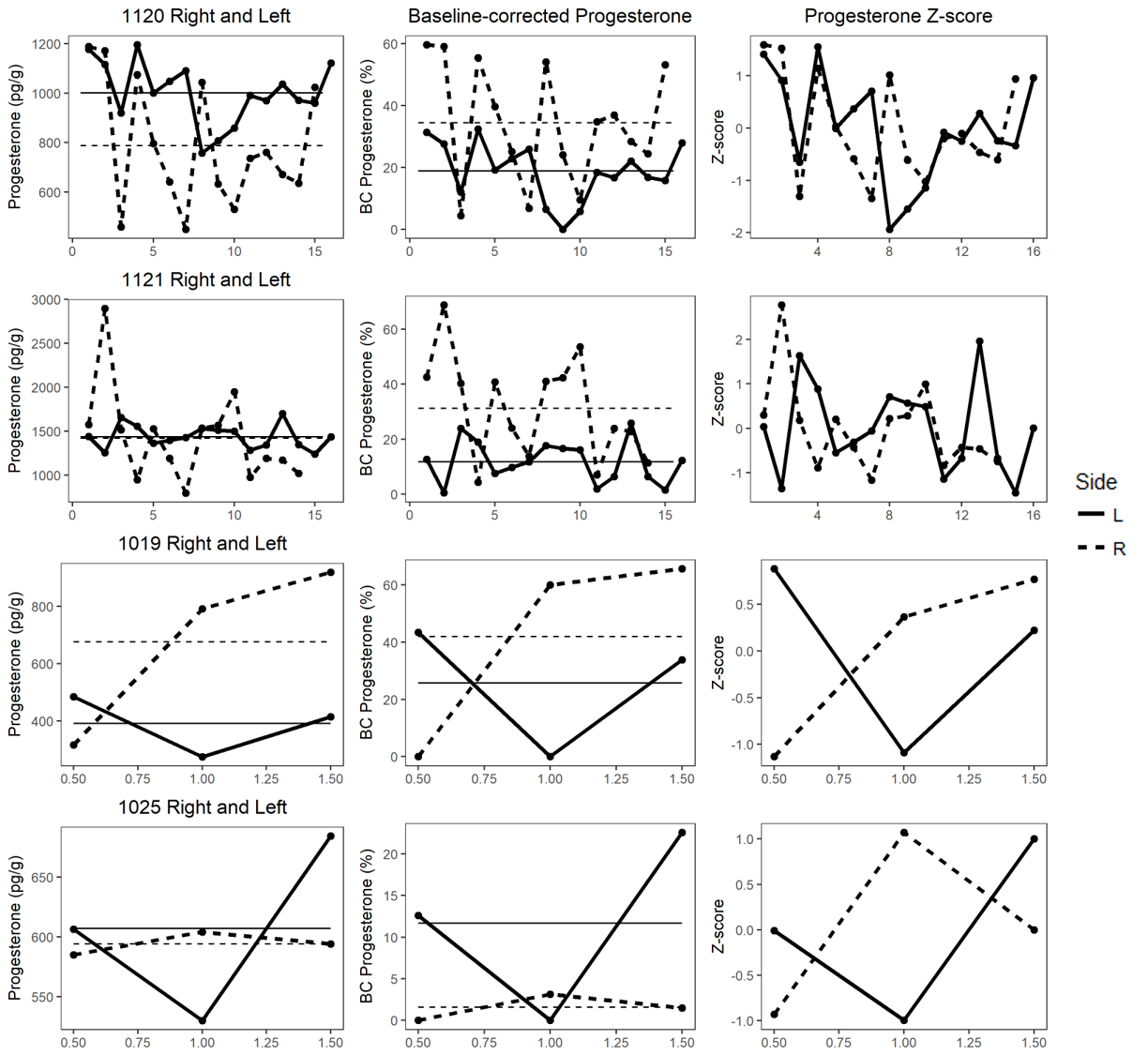

Supplement: suppl_data_coaa055 [file suppl_data_coaa055.zip › FigS4.png]

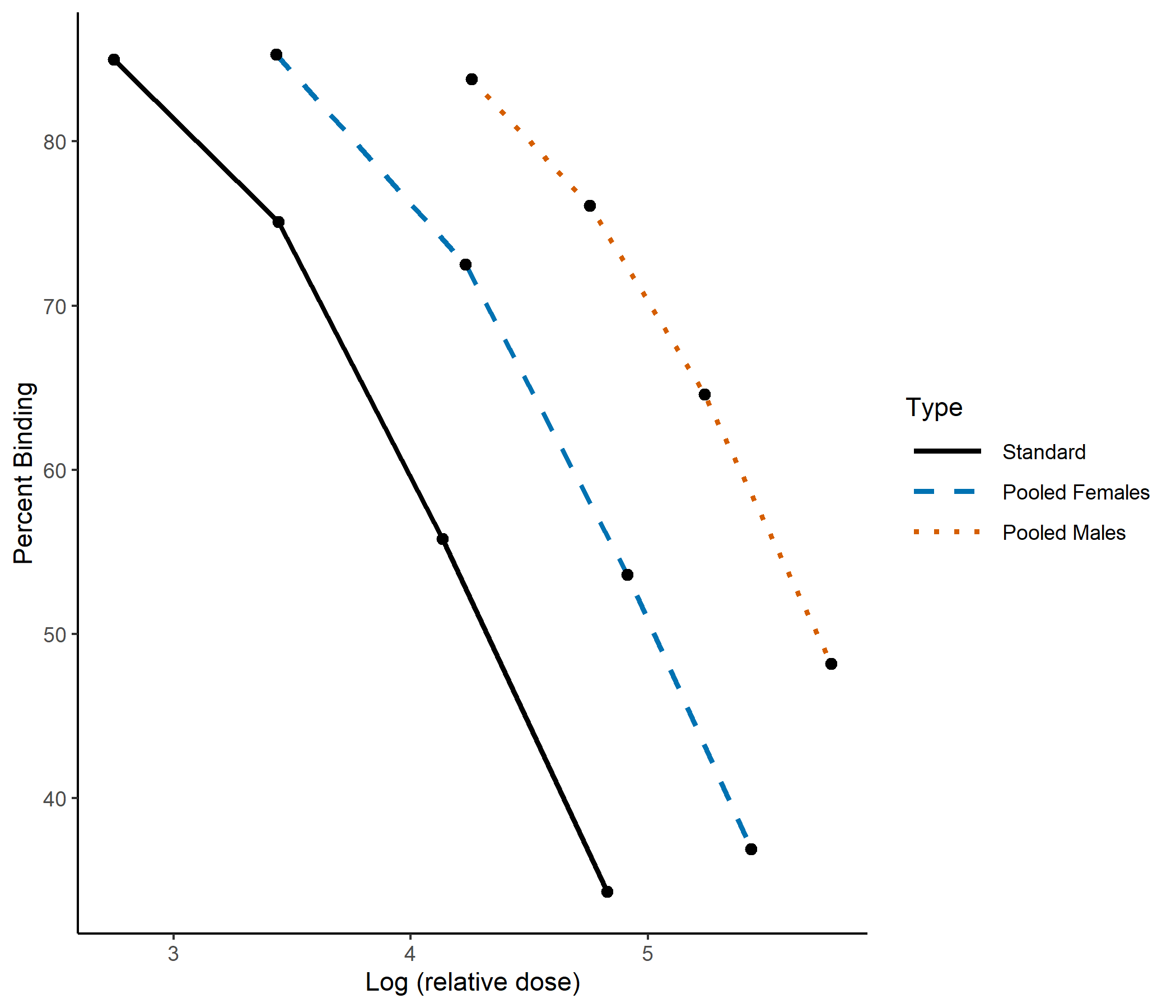

Supplement: suppl_data_coaa055 [file suppl_data_coaa055.zip › ProgVal_Parallelism.png]

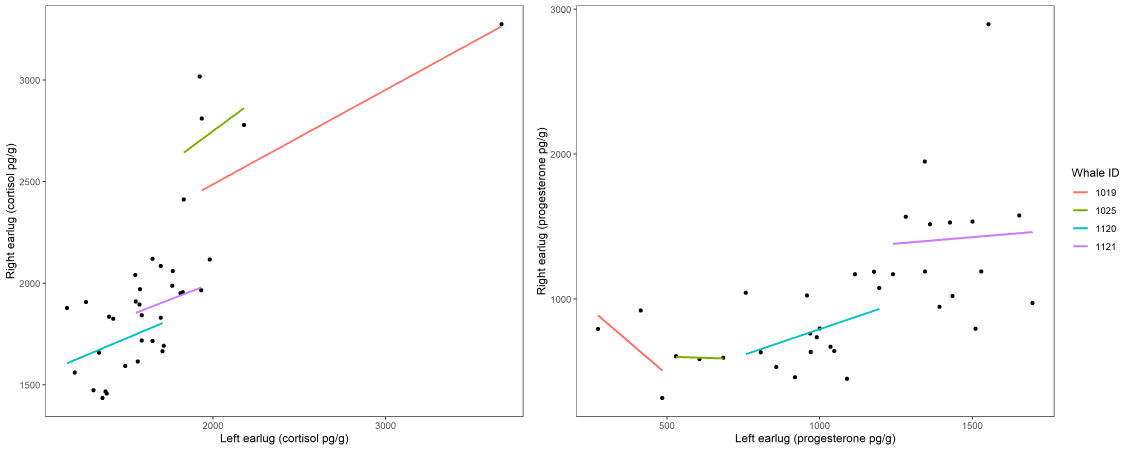

Supplement: suppl_data_coaa055 [file suppl_data_coaa055.zip › supfig_scatter.png]
